# Phytochemical Analysis and Biological Investigation of *Nepeta juncea* Benth. Different Extracts

**DOI:** 10.3390/plants9050646

**Published:** 2020-05-19

**Authors:** Majid Sharifi-Rad, Francesco Epifano, Serena Fiorito, José M. Álvarez-Suarez

**Affiliations:** 1 Department of Range and Watershed Management, Faculty of Water and Soil, University of Zabol, Zabol 98613-35856, Iran; 2 Dipartimento di Farmacia, Università “Gabriele d’Annunzio” Chieti-Pescara, Via dei Vestini 31, 66100 Chieti Scalo (CH), Italy; fepifano@unich.it (F.E.); serena.fiorito@unich.it (S.F.); 3 King Fahd Medical Research Center, King Abdulaziz University, Jeddah 21589, Saudi Arabia; 4 Grupo de Investigación en Biotecnología Aplicada a Biomedicina (BIOMED). Universidad de Las Américas, Quito 170125, Ecuador

**Keywords:** biological activities, bioactive compounds, human cancer cells, *Nepeta juncea* Benth

## Abstract

This study was carried out to screen the amount and the classes of secondary metabolites and to evaluate the antioxidant, cytotoxic, antifungal, and antibacterial activities of the methanolic, ethanolic, and water extracts of the roots, leaves, and flowers of *Nepeta juncea* Benth. The results show that the highest total phenol (69.54 ± 0.31 mg gallic acid equivalents (GAE)/g dry weight), total flavonoid (41.37 ± 0.17 mg quercetin equivalents (QE)/g dry weight), anthocyanin (6.52 ± 0.21 mg cyanidin/100 g dry weight), and tannin (47.36 ± 0.33 mg catechin/g dry weight) concentrations were recorded in the methanolic extract of the leaves of *N. juncea*. The gas chromatography–mass spectrometry (GC–MS) analysis of the extracts showed that 1,8-cineole, 4aα-7α-7aα-nepetalactone, β-pinene, terpinen-4-ol, and α-terpineol were the major compounds, respectively. The best 2, 2-diphenyl-1-picrylhydrazyl (DPPH) radical scavenging and ferric-reducing antioxidant, cytotoxic, antifungal, and antibacterial activities were observed for the methanolic extract of the leaves. For the two latter activities, the best activity was revealed on *Staphylococcus aureus*, *Bacillus cereus*, and *Candida albicans*. The minimum inhibitory concentration (MIC) values for the antimicrobial of the methanolic extract from the leaves were in the range of 25–100 µg/mL, whereas the minimum bactericidal concentration (MBC) values were in the range of 50–200 µg/mL. The results reported herein show that, for the first time in the literature, *N. juncea* is a remarkable source of antioxidant, antifungal, and antibacterial compounds.

## 1. Introduction

Plants have represented an important source of bioactive compounds (e.g., phenolics, terpenoids, aromatic components, essential oils, sterols, alkaloids, polysaccharides, tannins, and anthocyanin) for centuries [1,2]. Natural compounds play a significant role in drug discovery and in the development of novel therapeutic entities [3]. In recent decades, much attention has been paid to investigating the antioxidant and antibacterial activities of medicinal plants [4,5,6]. It has been proven that the antioxidant properties of medicinal plant products are mainly attributed to the above-mentioned phytochemicals [7]. These natural antioxidants prevent the destructive effects induced by oxidative stress of reactive oxygen species (ROS) [8,9], which are well known to be implicated in aging [10] and many acute and chronic diseases such as diabetes [11], cancer [12], and neurodegenerative disorders [13]. On the other hand, bacterial resistance to synthetic and semi-synthetic antibiotics is a rapidly increasing problem [14]. In addition, these antibiotics cause different adverse drug reactions such as immuno-suppression and hypersensitivity [15]. To overcome this problem, it is vital to find new antimicrobial agents that are not only able to suppress bacterial infections but are also able to have a long-lasting effect by boosting immune functions [16,17]. Likewise, multidrug drug resistance of cancer cells can lead to chemotherapy failure during the course of cancer treatment [18]. Thus, the use of phytotherapeutics is a promising anticancer method with fewer side effects than conventional medicines and is also an interesting strategy to prevent contaminations and infections in medicine and food products [19].

Candidiasis is the most common fungal infection. *Candida glabrata* and *Candida albicans* are two species that are usually implicated in the clinical picture. The candidiasis spectrum is vast, from mild symptoms such as the colonization of mucosal tissue to systemic pictures, with the invasion of various organs [20]. These yeasts are common microbiota and can become pathogenic in cases such as acquired or congenital immunodeficiency and immunosuppression due to severe stress [21]. Many extract types have been extensively studied in search of alternative therapies to combat these infections, e.g., *Mentha longifolia* (L.) Huds. [22], *Malva sylvestris* L. and *Psidium guajava* L. [23], and *Satureja intermedia* C.A.Mey [19].

The *Nepeta* genus is widely used in traditional medicine and is commonly applied for its anti-Alzheimer, anti-seizure, anti-nociceptive, memory enhancing, neuroprotective, antidepressant, and anti-infective effects in Iranian folk medicine [24]. It belongs to the Lamiaceae family, subfamily Nepetoideae, tribe Mentheae. *Nepeta* species are widely distributed across North America, Europe, Africa, and Asia; about 76 species of the genus *Nepeta* are found in Iran and Turkey, 58 in Pakistan, and 35 species in Western Himalaya [24]. Many biological activities have been reported for *Nepeta* spp., such as antioxidant, antibacterial, antifungal, anti-inflammatory, insecticidal, analgesic, and antidepressant activities, among others [24,25]. According to the phytochemical composition, this genus can be considered as two groups: the first group contains a high percentage of nepetalactone and its isomers, and, in the second group, 1,8-cineole and/or linalool are the main compounds [26]. Nepetalactones are the main compound (50–95%) in *N. cataria*, *N. caesarea*, *N. racemosa*, *N. argolica*, *N. sibirica*, *N. elliptica*, *N. x faasenii*, *N. rtanjensis*, *N. meyeri*, *N. nepetella*, *N. saccharata*, *N. coerulea*, and *N. parnassica.* A medium percentage of the nepetalactones (14–50%) is reported for *N. betonicifolia*, *N. grandiflora*, *N. spruneri, N. persica* and *N. crispa.* Finally, there are species that have minor percentage of the nepetalactones (0.5–7%) such as *N. pogonosperma*, *N. leucolaena*, and *N. sulfuriflora.* In these species, 1,8-cineole is the main compound [24]. *N. cataria* is one of the most investigated species of this genus, the results of which confirm its traditional applications [24]. Recent studies have reported promising activities for the *Nepeta binaludensis* Jamzad and *Nepeta satureioides* Boiss extracts, e.g., inhibition of melanogenesis and antioxidant activity [24,27]. The main purpose of this study was to investigate the phytochemical composition and to assess the antioxidant, cytotoxic, antifungal, and antibacterial activities of the methanolic, ethanolic, and water extracts of the roots, leaves, and flowers of *N. juncea* Benth. To the best of our knowledge, there are no systematic studies on the in vitro antioxidant, cytotoxic, antifungal, and antibacterial activities of *N. juncea* Benth.

## 2. Results and Discussion

### 2.1. Total Phenol Concentration

The results of the total phenolic content determination of the *N. juncea* extracts are shown in Table 1. In all of the extracts, the total phenolic content was higher in the leaves extracts than in those from the roots and flowers. The highest content of total phenol was measured in the methanolic extract for each part of *N. juncea,* followed by the ethanol and water extracts, respectively. The methanolic extract of the leaves had the highest value of total phenol content (69.54 ± 0.31 mg gallic acid equivalents (GAE)/g dry weight), and the lowest total phenolic content (13.46 ± 0.26 mg GAE/g dry weight) was observed in the water extract of the roots. The variation in the total phenolic content in the different extracts is related to the different solubility of the phenolic compounds; this change in solubility may be driven by the polarity of the solvent [28]. This is in line with the results of previous literature showing how methanol behaves as a better extraction solvent for phenolic compounds [29,30].

### 2.2. Total Flavonoid Concentration

The results of the total flavonoid content determination of the *N. juncea* extracts are shown in Table 1. The methanolic extract of the leaves showed higher values of flavonoid content (41.37 ± 0.17 mg quercetin equivalents (QE)/g dry weight) than the other extracts under investigation. On the contrary, the flavonoid concentration of the water extract of the roots was the lowest (5.23 ± 0.35 mg QE/g dry weight).

### 2.3. Total Anthocyanin Concentration

As shown in Table 1, the maximum total anthocyanin content (6.52 ± 0.21 mg cyanidin/100 g dry weight) was observed in the methanolic extract of the leaves, and the minimum (1.51 ± 0.14 mg cyanidin/100 g dry weight) was recorded in the ethanolic extract of the roots. Comparing the solvents, the anthocyanin concentration followed the order of methanol > water > ethanol, meaning that methanol is the best solvent for anthocyanin extraction from *N. juncea*. This result is in accordance with previous findings [31,32].

### 2.4. Total Tannin Concentration

The results of the total tannin content determination of the *N. juncea* extracts are shown in Table 1. Among the three extracts, the methanolic extract of the leaves showed the maximum tannin content (47.36 ± 0.33 mg catechin/g dry weight), and the ethanolic extract of the roots showed the minimum tannin content (10.21 ± 0.26 mg catechin/g dry weight). It is reported that the yields of extraction increase with the polarity of the solvent [33]; accordingly, maximum extraction yields are usually achieved using methanol or water as a solvent [34]. Comparing the different plant parts, the tannin content followed the order of leaves > flowers > roots for each solvent.

### 2.5. Gas Chromatography–Mass Spectrometry Analysis

**Figure 1:**
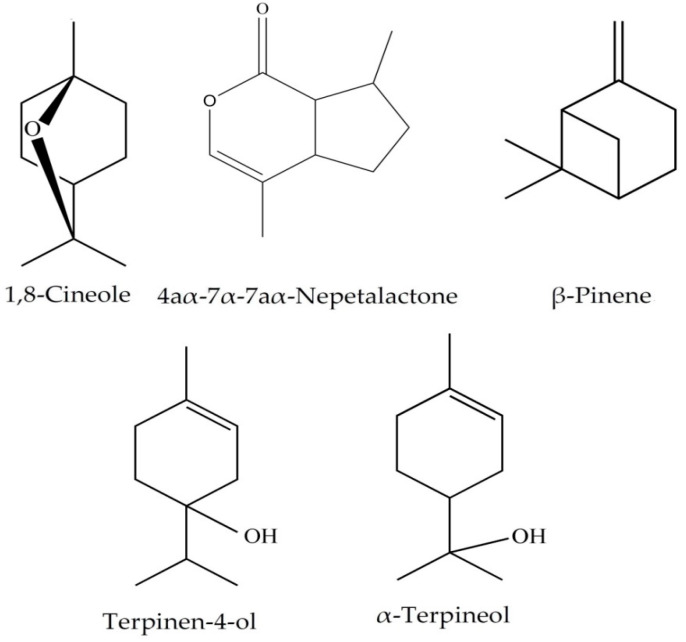
Chemical structure of the major compounds of the *Nepeta juncea* extracts.

The chemical composition of the *N. juncea* extracts is shown in Table 2. The main constituents were 1,8-Cineole, 4aα-7α-7aα-Nepetalactone, β-Pinene, Terpinen-4-ol, and α-Terpineol, respectively (Figure 1). Several reports have described the antioxidant and the antimicrobial activities of these compounds [35,36,37,38]. Shafaghat and Khodamali [37] analyzed the leaf oil of *N. persica,* in which 4aβ,7α,7aβ-Nepetalactone (62.3%), 4aα,7α,7aβ-Nepetalactone (28.3%), and β-ocimene (3.6%) were the major components, followed by α-pinene (1.8%). The essential oils isolated from the different parts of *N. sintenisii* Bornm. (i.e., flower, leaf, stem, and root) were analyzed by GC and GC–MS. 4aβ,7α,7aβ-Nepetalactone was characterized in the flower (60.3%), leaf (34.6%), stem (64.2%), and root (61.2%) as the main constituent, and the highest and lowest amounts of nepetalactone isomers were observed in the flower and root, respectively [39].

### 2.6. Radical Scavenging Activity

**Figure 2:**
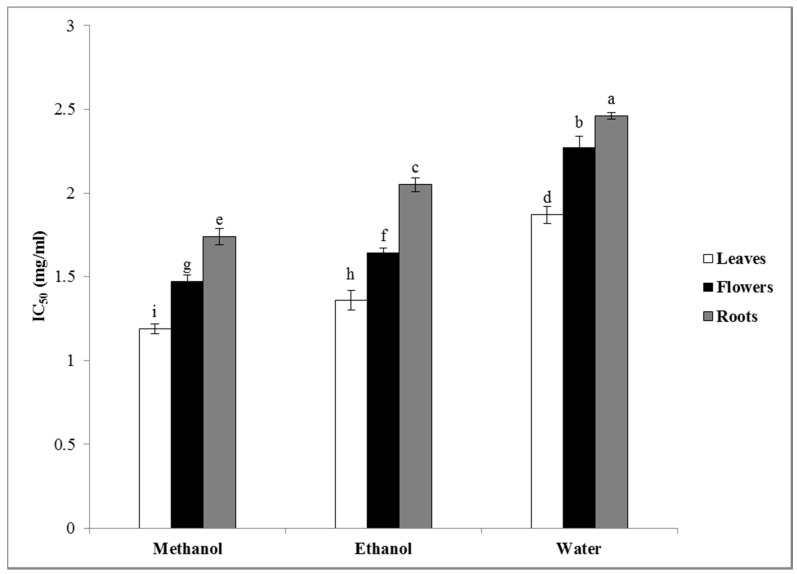
DPPH scavenging activity (expressed as IC_50_ (50% inhibitory concentration)) of the different extracts of *Nepeta juncea*. Columns belonging to the same dataset and labeled with different letters are significantly different, *p* < 0.05 (*n* = 3).

The free radical scavenging properties of the extracts were determined by the 2,2-diphenyl-1-picrylhydrazyl (DPPH) assay (Figure 2). The methanolic extract of the leaves had the maximum and the water extract of the roots had the minimum antiradical activities. It was observed that the methanolic extract of *N. juncea* had the highest activity, followed by the ethanolic and water extracts, respectively. For each solvent, the antioxidant activity decreased according to the following order: leaves > flowers > roots. In this study, as in many other studies [40,41], a direct relationship between antioxidant activity and total phenolic and flavonoid content was observed. The results show that the IC_50_ (50% inhibitory concentration) of the different extracts varied between 1.19 ± 0.03 and 2.46 ± 0.02 mg/mL.

### 2.7. Ferric Reducing Antioxidant Power (FRAP) Assay

**Figure 3:**
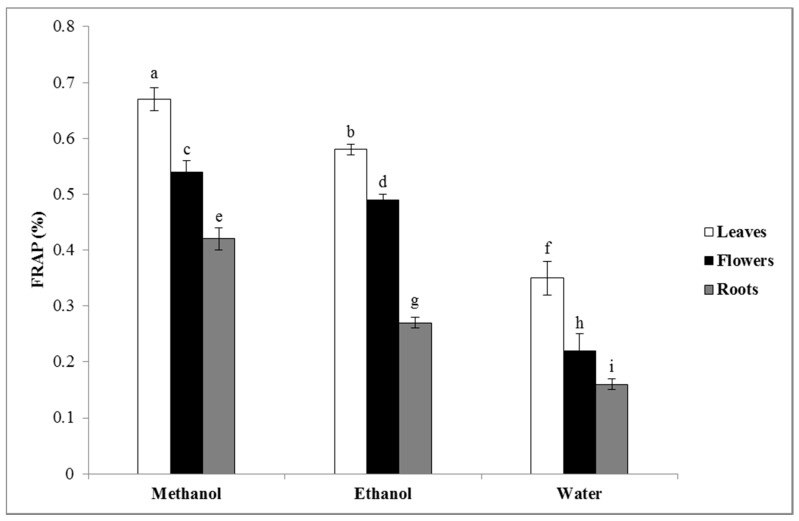
Ferric reducing antioxidant power (FRAP) values of the different extracts of *Nepeta juncea*. Columns belonging to the same dataset and labeled with different letters are significantly different, *p* < 0.05 (*n* = 3).

Figure 3 shows the antioxidant activity of the extracts using the FRAP assay. According to the results, the methanolic extract of the leaves was the most active extract in the FRAP assay, and the water extract of the roots showed the lowest antioxidant activity. In many studies, it has been reported that there is a direct correlation between the antioxidant activity and the content of phenolics, flavonoids, anthocyanin, and tannins of plant extracts [42,43,44]. According to the results, the highest value of phenolic, flavonoid, tannin, and anthocyanin contents was recorded in the methanolic extract of the leaves.

### 2.8. Cytotoxicity Activity

The cytotoxicity activity results are summarized in Table 3. The *N. juncea* extracts exhibited a dose-dependent reduction in the survival of both cancer cells. According to the results, the methanolic extracts of *N. juncea* had the maximum cytotoxicity activity in both cancer cell lines, while the minimum cytotoxicity activity was observed for the water extracts. Skorić et al. [45] studied the cytotoxicity of *Nepeta rtanjensis* toward the HeLa, K562, A549, LS-174, and MDA-MB-231 cancer cell lines. They reported that the application of *N. rtanjensis* essential oil led to the emergence of morphological changes in the investigated cancer cell lines, and thus suggested that this oil may be applied as a potential anticancer therapy.

### 2.9. Antifungal Activity

From the results shown in Table 4, the best antifungal activity against the tested fungi was observed in the *N. juncea* methanolic extracts with minimum inhibitory concentration (MIC) values ranging from 25 to 100 µg/mL. *Candida albicans* was more sensitive with an MIC value of 25, 50, and 50 µg/mL for the methanolic extracts of the leaves, flowers, and roots, respectively. The minimum fungicidal concentration (MFC) was in the range of 50–100 µg/mL for the methanolic extracts. The antifungal activities of the essential oils isolated from other *Nepeta* species have been reported elsewhere [46,47]. According to the results of the current study, the main compounds of the *N. juncea* extracts were 1,8-cineole and 4aα-7α-7aα-Nepetalactone. Regarding previous studies, these compounds could be considered as the active components responsible for the extracts’ antifungal activities [48,49].

### 2.10. Antibacterial Activity

#### Determination of the Minimum Inhibitory Concentration (MIC) and the Minimum Bactericidal Concentration (MBC)

The results in Table 5 show that the best antibacterial activity against the tested bacteria was from the *N. juncea* methanolic extracts with MIC values ranging from 25 to 100 µg/mL. Among them, *S. aureus* and *B. cereus* were the most sensitive bacteria with an MIC value of 25, 25, and 50 µg/mL for the methanolic extracts of the leaves, flowers, and roots, respectively. The results show that the MBC was in the range of 50–200 µg/mL for the methanolic extracts. According to the results, the extracts had lower MIC and MBC values against all tested Gram-positive bacteria compared to Gram-negative bacteria. The lower MIC and MBC values indicate the higher antibacterial activity of the extracts on the tested bacteria strains [50].

## 3. Materials and Methods

### 3.1. Preparation of the Plant Extracts

The roots, leaves, and flowers of *N. juncea* Benth. were collected from the Saravan rangelands, Sistan, and Baluchesrtan, Iran. The genus and species of this plant were identified at the Department of Botany, Shahid Beheshti University of Medical Sciences, Tehran, Iran, where a voucher specimen (No. 842) was deposited. The different plant parts were dried in an oven at 40 °C for 72 h and then ground to a fine powder using an electric grinder (Pars Khazar, Tehran, Iran). The plant extracts were obtained by magnetic stirring of 2.5 g of powdered dry matter with 50 mL of solvent (methanol, 96% ethanol, and water) for 40 min at room temperature (24 ± 2 °C). The extracts were kept for 24 h at 4 °C, filtered through Whatman filter paper No. 1, and the filtrate evaporated to complete dryness under vacuum. A stock solution of different extracts (1 mg/mL) dissolved in methanol was used for the experiments.

### 3.2. Total Phenol Concentration

Total phenol concentration was determined using the Folin–Ciocalteu reagent, as described by Dewanto et al. [51]. Briefly, an aliquot of the diluted extract was added to 0.5 mL of distilled water and then completely mixed with 0.125 mL of the Folin–Ciocalteu reagent. After 3 min, 1.25 mL of 5% (*w/v*) of Na_2_CO_3_ was added and the resulting solution mixed thoroughly. For adjusting the final volume to 4 mL, distilled water was added. Finally, the resulting mixture was kept in darkness at room temperature for 2 h, and then the absorbance of the mixture was recorded at 760 nm using a UV-Vis spectrophotometer (UV-1800 240 V, Shimadzu Corporation, Kyoto, Japan). The total phenol value is represented as milligrams of gallic acid equivalents per gram of dry weight (mg GAE/g dry weight). A standard calibration curve was drawn at the same operating conditions using gallic acid (20–200 μg/mL, y = 0.0089x – 0.0003, R^²^ = 0.992) as a positive control.

### 3.3. Total Flavonoid Concentration

The total flavonoid content was determined based on the colorimetric assay described by Chang et al. [52] with minor modifications. In summary, 0.5 mL of each extract was separately mixed with 1.5 mL of methanol, 0.1 mL of potassium acetate (1 mol/L), 0.1 mL of AlCl_3_ (10%), and 2.8 mL of distilled water. The resulting mixture was kept at room temperature for 30 min. The absorbance of the mixture was recorded at 415 nm by using a UV-Vis spectrophotometer. Quantitative determination of the flavonoid content was calculated as quercetin from a calibration curve. Quercetin was used as the standard (10–100 μg/mL, y = 0.0092x – 0.034, R^²^ = 0.996), and the results are expressed as milligrams of quercetin equivalents per gram of dry weight (mg QE/g dry weight).

### 3.4. Total Anthocyanin Concentration

The total anthocyanin concentration was measured based on the pH differential method explained by Vega-Arroy et al. [53] with minor modifications. Ten milliliters of each extract were mixed with hydrochloric acid (1 M) or sodium hydroxide (1 M) to reach a pH of 1 or 4.5. The absorbance was recorded using a UV-Vis spectrophotometer at 520 and 700 nm. Cyanidin-3-glucoside was used as the standard (5–50 μg/mL, y = 0.0201x + 0.0168, R^²^ = 0.991). The concentration of total anthocyanin was determined as cyanidin-3-glucoside equivalents (mg/100 g) using Equations (1) and (2):

A = (A520 nm − A700 nm) pH 1.0 − (A520 nm − A700 nm) pH 4.5(1)

Total anthocyanin (mg/100 g) = (A × MW × DF × 1000)/ε × 1(2)

where A is the difference in absorbance; MW (molecular weight) = 449.2 g/mol for cyanidin-3-glucoside; DF is the dilution factor; 1 = quartz cell pathway (1 cm); and ε is the molar extinction coefficient for cyanidin-3-glucoside (26,900 M^−1^ cm^−1^).

### 3.5. Total Tannin Concentration

The total tannin concentration was determined based on the method of Sun et al. [54]. In brief, 2 mL of vanillin solution (4%) in methanol and 1.5 mL of concentrated HCl were added to 50 μL of the diluted sample. After 25 min, the absorption of the reaction mixture was determined at 500 nm using a UV-Vis spectrophotometer. Methanol was used as the blank, and catechin was used as the standard (20–120 μg/mL, y = 0.0036x + 0.0011, R^²^ = 0.994). The total condensed tannin content is represented as mg (+)-catechin/g dry weight. 

### 3.6. Gas Chromatography–Mass Spectrometry Analysis

Gas chromatography–mass spectrometry analysis was carried out on a GCMS-QP2010 system (Shimadzu, Tokyo, Japan). Hexane (≥99%; Sigma–Aldrich, Germany) was used to dilute 20 μL of each extract to 1 mL. The used column was Rtx-5MS (Restek, Bellefonte, PA, USA) (30 m × 0.25 mm i.d. × 0.25 µL film thickness). The helium flow rate (99.999%; AGA Lithuania) carrier gas was adjusted at 1.23 mL/min. After injection, the temperature of the oven was retained at 40 °C for 2 min; then, it was programmed to increase by 3 °C/min until it reached 210 °C, at which time the column was retained for 10 min. The ratio of the split was 1:10. Detection was performed by 70-eV electron ionization. The compounds were identified using the mass spectra library (NIST 14) and the similarities of the mass spectra with the mass spectral data from the literature [55].

### 3.7. Antioxidant Activity

The antioxidant activity of the extracts was quantified as radical scavenging capacity against 2,2-diphenyl-1-picrylhydrazyl radical (DPPH) following the method of Sharifi-Rad et al. [56]. Different concentrations of extracts (5, 10, 50, and 100 μg/mL) were added to 0.5 mL of 0.2 mmol L^−1^ DPPH–methanol solution and left at room temperature (25 ± 2 °C) for 45 min. The absorbance of the resulting solution was recorded using a UV-Vis spectrophotometer at 517 nm. The percentage inhibition of the free radical DPPH was calculated using Equation (3).

Antioxidant Activity (%) = (A_blank_ – A_sample_/A_blank_) × 100 (3)

where A_blank_ is the absorbance of the control (consisting of the solvent and DPPH) and A_sample_ is the absorbance in the presence of the plant extract. Ascorbic acid solutions (5, 10, 50, and 100 μg/mL) were used as standard and the IC_50_ values were calculated from the percent inhibition. The results are expressed as IC_50_ values (e.g., the concentration of the extract required to scavenge 50% of the DPPH radical).

### 3.8. Ferric Reducing Antioxidant Power (FRAP) Assay

The FRAP assay was performed based on the method explained previously [57]. To prepare the FRAP reagent, 300 mM sodium acetate buffer (pH 3.6, 10 mL) was added to 20 mM iron (III) chloride (1 mL) and 10 mM TPTZ (2,4,6-tripyridyl-S-triazine) solution in 40 mM hydrochloric acid (1 mL). This reagent was used in a water bath at 37 °C. The sample (20 µL) was mixed with the FRAP reagent (150 µL). The absorbance was immediately recorded at 593 nm. The FRAP value was determined using Equation (4).

FARP value (%) = [(A_s_ − A_b_) / (A_c_ − A_b_)] × 2 (4)

where A_s_ is the absorbance of the sample; A_b_ is the absorbance of the blank, reacted with distilled water (20 µL) and FRAP reagent (150 µL); and A_c_ is the absorbance of the positive control, reacted with ascorbic acid (20 µL) and the FRAP reagent (150 µL).

### 3.9. Cytotoxicity Activity

#### 3.9.1. Human Cancer Cell Lines

The human breast adenocarcinoma (MCF-7) cells (ATCC® HTB22™) and the human hepatocellular carcinoma (Hep-G_2_) cell line (ATCC® HB8065™) were purchased from the American Type Culture Collection (ATCC, Rockville, MD, USA). The cells were cultivated in Dulbecco’s modified Eagle’s Medium accompanied by L-glutamine (2%), HEPES (*N*-2-hydroxyethylpiperazine-*N*-2-ethane sulfonic acid) buffer, heat-inactivated fetal bovine serum (10%), and 40 μg/mL gentamicin (Sigma-Aldrich, St. Louis, MO, USA). The cells were kept at in a humidified atmosphere with CO_2_ (5%) at 37 °C and were sub-cultured four times a week.

#### 3.9.2. Cytotoxicity Assay

The cytotoxicity of *N. juncea* extracts toward the cancer cells was investigated using the crystal violet staining method as explained previously [58]. Briefly, 96-well tissue culture microplates were used for the incubation of the cells (1 × 10^4^ cells per well supplemented with 100 μL of growth medium). Various concentrations of the *N. juncea* extracts (0, 25, 50, 100, and 200 μg/mL) were added after 24 h of seeding at 37 °C. Two-fold serial dilutions of the extracts were added to confluent cell monolayers into the 96-well microtiter plates. The incubation of the plates was performed at 37 °C for 48 h in a humidified incubator with CO_2_ (5%). The viable cells were measured by the colorimetric method. In brief, the medium was aspirated and crystal violet solution in methanol (2% *v/v*) was added to each well. Afterward, 0.2 mL of glacial acetic acid–ethanol solution (1.0 mL glacial acetic acid per 100 mL 70% ethanol) was added to each well and mixed completely. The absorbance was measured using an automatic microplate reader at 595 nm. Vinblastine sulfate (0, 25, 50, 100, and 200 μg/mL) was considered as a standard anticancer drug.

### 3.10. Antifungal Activity

#### 3.10.1. Strains and Media

The antifungal activity of the *N. juncea* extracts was evaluated on oral pathogens, including *Candida albicans* (ATCC 13803) and *C. glabrata* (ATCC 90030). The strains were cultured under constant shaking (200 rpm) at 30 °C in yeast-extract peptone dextrose (YPD) liquid medium consisting of 1% (*w/v*) yeast extract, 2% (*w/v*) peptone, and 2% (*w/v*) dextrose.

#### 3.10.2. Antifungal Susceptibility Test

The antifungal susceptibility test was carried out on the strains based on the broth microdilution procedure, as explained by Quan et al. [59]. The initial concentration of fungi suspended in RPMI 1640 media (Sigma, St. Louis, MO, USA) was about 10^3^ cells/mL, and the initial concentration of the *N. juncea* extracts ranged from 25 to 200 µg/mL. The wells that included fungi inoculum without any extracts were considered as negative control, and fluconazole (2–200 µg/mL) was used as a reference or positive control. The 96-well plates were incubated for 24–48 h at 35 °C. The minimum inhibitory concentrations (MICs) were determined using optical density. Finally, 100 μL of the culture from each well showing no visible growth was sub-cultured on Sabouraud dextrose agar (Merck, Darmstadt, Germany) to measure the minimum fungicidal concentrations (MFCs).

### 3.11. Antibacterial Activity

#### 3.11.1. Bacterial Strain Preparation

Different American-Type Cell Culture (ATCC) reference bacterial strains, including *Staphylococcus aureus* (ATCC: 25923), *Bacillus cereus* (ATCC: 11778), *Escherichia coli* (ATCC: 25922), and *Shigella flexneri* (ATCC: 12022), were obtained from the Iranian microbial collections of the Pasteur Institute of Iran. The bacterial strains were incubated for 24 h at 37 °C on nutrient broth. All bacteria strains were adjusted to 0.5 McFarland standard by the optical density (OD) method at 620 nm (1.5 × 10^8^ CFU/mL), as described by Sharifi-Rad et al. [60].

#### 3.11.2. Determination of the Minimum Inhibitory Concentration (MIC)

The micro-broth dilution method was used to measure the minimum inhibitory concentrations (MICs) of the extracts against the tested bacteria, as recommended by the Clinical and Laboratory Standards Institute [61]. The concentrations of the extracts considered for MICs ranged from 25 to 200 µg/mL. The test was carried out using polystyrene 96-well plates. Two-fold serial dilutions of the extracts were prepared in cation-adjusted Mueller–Hinton broth. Then, 50 µL of Mueller–Hinton broth and 50 µL of the different concentrations of the extracts were used for preparing each inoculum. The starting inoculum for each strain was 1.5 × 10^8^ CFU/mL, and the wells that included bacterial inoculum without any extracts were considered as the control. Plates were incubated for 24 h at 37 °C. The lowest concentration of the extracts at which the microorganisms showed no visible growth was considered as the MIC.

#### 3.11.3. Determination of the Minimum Bactericidal Concentration (MBC)

Determination of the MBC values was performed based on a method described by the Clinical and Laboratory Standards Institute [61]. After 24 h of incubation, 100 μL of the culture from each well of the micro-broth test was sub-cultured on Mueller–Hinton agar plates, which were further incubated for 24 h at 37 °C. The MBC was defined as the lowest concentration of the extracts at which there was no sign of bacterial growth.

### 3.12. Statistical Analysis 

All experiments were performed in triplicate. Data were analyzed by the statistical software package SPSS v 11.5 (IBM Corporation, Armonk, NY, USA). The analysis of variance (ANOVA) and Duncan’s multiple range methods were used to compare any significant differences between samples and solvents. The results are presented as means values ± standard deviations (SD).

## 4. Conclusions

The leaf extracts of *N. juncea* showed higher antioxidant, cytotoxic, antifungal, and antibacterial activities than the flower and root extracts, and the methanolic extracts of the leaves had the highest of these activities. This extract also showed high phenolic, flavonoid, anthocyanin, and tannin contents. It could be hypothesized that these compounds may be responsible for the extract’s biological activities. To the best of our knowledge, this is the first report on the antioxidant, cytotoxic, antifungal, and antibacterial activities of *N. juncea* extracts. The results of the current study confirm that *N. juncea* has antioxidant, cytotoxic, antifungal, and antibacterial activities and that it may be appropriate as a phytopharmaceutical ingredient.

## Figures and Tables

**Table 1:** The total phenols, total flavonoids, anthocyanin, and tannin concentrations in the different extracts of *Nepeta juncea.*

Solvent	Plant Part	Total Phenols (mg GAE/g Dry Weight)	Total Flavonoids (mg QE/g Dry Weight)	Anthocyanin (mg Cyanidin/100 g Dry Weight)	Tannin (mg Catechin/g Dry Weight)
Methanol	Leaves	69.54 ± 0.31 ^a^	41.37 ± 0.17 ^a^	6.52 ± 0.21 ^a^	47.36 ± 0.33 ^a^
	Flowers	45.61 ± 0.14 ^c^	26.42 ± 0.31 ^c^	4.35 ± 0.34 ^c^	32.16 ± 0.21 ^c^
	Roots	21.33 ± 0.46 ^g^	9.62 ± 0.15 ^g^	2.89 ± 0.42 ^f^	23.15 ± 0.15 ^f^
Ethanol	Leaves	52.36 ± 0.27 ^b^	34.23 ± 0.29 ^b^	3.42 ± 0.43 ^d^	28.14 ± 0.35 ^d^
	Flowers	30.22 ± 0.14 ^e^	19.81 ± 0.53 ^e^	2.45 ± 0.28 ^g^	19.31 ± 0.14 ^g^
	Roots	18.12 ± 0.28 ^h^	7.16 ± 0.26 ^h^	1.51 ± 0.14 ^i^	10.21 ± 0.26 ^i^
Water	Leaves	33.17 ± 0.34 ^d^	23.25 ± 0.49 ^d^	4.63 ± 0.27 ^b^	36.21 ± 0.24 ^b^
	Flowers	24.71 ± 0.12 ^f^	14.32 ± 0.13 ^f^	3.11 ± 0.18 ^e^	24.17 ± 0.16 ^e^
	Roots	13.46 ± 0.26 ^i^	5.23 ± 0.35 ^i^	1.96 ± 0.36 ^h^	16.11 ± 0.52 ^h^

The mean values within the columns that share different letters are significantly different, *p* < 0.05 (*n* = 3). GAE, gallic acid equivalents; QE, quercetin equivalents.

**Table 2:** Chemical composition of the *Nepeta juncea* extracts.

Compound	RI	Molecular Formula	Methanolic Extract			Ethanolic Extract			Water Extract		
			Leaves (%)	Flowers (%)	Roots (%)	Leaves (%)	Flowers (%)	Roots (%)	Leaves (%)	Flowers (%)	Roots (%)
α-Thujene	924	C_10_H_16_	0.7	0.4	0.2	0.4	0.3	0.2	0.2	0.2	0.1
α-Pinene	935	C_10_H_16_	1.8	0.9	0.3	1.2	0.7	0.1	0.7	0.3	0.1
Sabinene	970	C_10_H_16_	0.2	-	-	0.1	-	-	-	-	-
β-Pinene	976	C_10_H_16_	4.3	1.6	0.5	2.7	0.9	0.3	1.1	0.5	0.2
Myrcene	984	C_10_H_16_	0.1	0.1	-	-	0.1	-	-	-	-
α-Terpinene	1014	C_10_H_16_	0.4	0.2	0.1	0.1	0.1	0.1	-	0.1	0.1
*p*-Cymene	1019	C_10_H_14_	0.5	0.2	0.1	0.2	0.1	0.1	0.1	0.1	-
1,8-Cineole	1032	C_10_H_18_O	41.6	20.2	4.1	24.3	15.4	2.2	11.2	8.7	1.4
γ-Terpinene	1053	C_10_H_16_	0.1	-	-	-	-	-	-	-	-
*cis*-Sabinene hydrate	1058	C_10_H_18_	-	0.1	-	-	0.1	-	-	0.1	-
Terpinolene	1080	C_10_H_16_	0.3	0.2	0.1	0.1	0.2	0.1	0.1	0.1	0.1
Linalool	1085	C_10_H_18_O	0.8	0.5		0.3	0.3		0.1	0.2	
*trans*-Sabinene hydrate	1088	C_10_H_18_O	0.3	-	0.1	-	-	0.1	0.1	-	-
*trans*-Pinocarveol	1127	C_10_H_16_O	-	0.1	-	-	0.1	-	-	-	-
Sabinol	1135	C_10_H_16_O	0.9	0.7	0.2	0.5	0.5	0.1	0.2	0.3	0.1
Pinocarvone	1142	C_10_H_14_O	0.5	0.4	0.1	0.4	-	0.1	0.1	-	0.1
Isopulegol	1145	C_10_H_18_O	0.6	0.7	-	0.5	0.5	-	0.3	0.2	-
Pinocamphone	1161	C_10_H_16_O	0.3	0.3	-	0.1	0.2	-	-	-	-
Terpinen-4-ol	1167	C_10_H_18_O	3.7	3.4	0.9	2.8	2.6	0.8	1.4	1.5	0.3
α-Terpineol	1177	C_10_H_18_O	2.3	1.9	0.8	1.3	0.9	0.5	0.8	0.6	0.2
Geraniol	1225	C_10_H_18_O	0.6	0.5	0.2	0.4	-	0.1	0.1	0.1	-
Geranial	1269	C_10_H_16_O	0.4	-	0.2	0.1	-	0.1	0.1	-	0.1
4aα-7α-7aα-Nepetalactone	1340	C_10_H_14_O_2_	16.2	18.4	9.3	10.1	12.4	7.6	4.3	5.3	3.7
4aα-7α-7aβ-Nepetalactone	1365	C_10_H_14_O_2_	0.8	0.9	0.4	0.3	0.5	0.1	0.1	0.3	0.1
4aβ-7α-7aβ-Nepetalactone	1367	C_10_H_14_O_2_	0.6	0.8	0.2	0.2	0.4	0.1	0.1	0.2	-
Geranyl acetate	1384	C_12_H_20_O_2_	0.1	-	0.1	-	-	0.1	-	-	-
β-Farnesene	1449	C_15_H_24_	0.1	-	-	0.1	-	-	0.1	-	-
Germacrene-d	1483	C_15_H_24_	0.3	0.1	-	0.2	0.1	-	0.1	-	-
*cis*-α-Bisabolene	1493	C_15_H_24_	-	0.1	-	-	-	-	-	-	-
α-Farnesene	1497	C_15_H_24_	0.2	-	0.2	0.1	-	0.1	0.1	-	0.1
Spathulenol	1575	C_15_H_24_O	0.1	0.1	-	-	-	-	-	0.1	-
Total identified compounds%			78.8	52.8	18.1	46.5	36.4	12.9	21.4	18.9	6.7

RI—retention index.

**Table 3:** Cytotoxic activity of *Nepeta juncea* extracts toward human cancer cell lines.

Solvent	Plant Part	Concentration (µg/mL)	Viability (%)	
			MCF-7	Hep-G2
Methanol	Leaves	25	86.2 ± 0.2	88.6 ± 0.4
		50	75.1 ± 0.5	76.3 ± 0.2
		100	62.7 ± 0.3	64.2 ± 0.3
		200	55.9 ± 0.1	58.4 ± 0.1
	Flowers	0	100	100
		25	94.3 ± 0.1	96.7 ± 0.5
		50	84.7 ± 0.2	86.4 ± 0.2
		100	70.2 ± 0.4	73.9 ± 0.1
		200	66.1 ± 0.2	68.3 ± 0.3
	Roots	0	100	100
		25	95.5 ± 0.5	98.9 ± 0.2
		50	93.2 ± 0.6	95.6 ± 0.4
		100	89.1 ± 0.4	92.4 ± 0.3
		200	87.4 ± 0.2	89.2 ± 0.2
Ethanol	Leaves	0	100	100
		25	91.2 ± 0.1	92.1 ± 0.3
		50	82.4 ± 0.3	84.3 ± 0.2
		100	70.3 ± 0.2	72.1 ± 0.5
		200	62.8 ± 0.5	65.6 ± 0.4
	Flowers	0	100	100
		25	95.9 ± 0.4	97.2 ± 0.1
		50	86.8 ± 0.2	89.2 ± 0.2
		100	72.7 ± 0.1	75.6 ± 0.1
		200	69.3 ± 0.4	71.3 ± 0.1
	Roots	0	100	100
		25	96.7 ± 0.3	99.2 ± 0.2
		50	94.2 ± 0.2	96.3 ± 0.1
		100	91.6 ± 0.6	94.2 ± 0.2
		200	89.2 ± 0.5	92.3 ± 0.6
Water	Leaves	0	100	100
		25	96.3 ± 0.7	98.4 ± 0.7
		50	92.5 ± 0.5	94.2 ± 0.3
		100	88.3 ± 0.3	91.1 ± 0.2
		200	85.2 ± 0.2	87.3 ± 0.4
	Flowers	0	100	100
		25	97.6 ± 0.5	99.1 ± 0.4
		50	96.2 ± 0.3	97.6 ± 0.6
		100	91.4 ±0.7	93.9 ± 0.1
		200	87.4 ± 0.3	89.6 ± 0.3
	Roots	0	100	100
		25	98.8 ± 0.2	99.7 ± 0.4
		50	96.2 ± 0.1	98.1 ± 0.2
		100	94.1 ± 0.5	96.3 ± 0.5
		200	93.8 ± 0.3	95.2 ± 0.1
Vinblastine		0	100	100
		25	51.6 ± 0.2	55.2 ± 0.3
		50	24.2 ± 0.1	19.5 ± 0.5
		100	9.4 ± 0.4	11.7 ± 0.2
		200	3.7 ± 0.3	4.6 ± 0.1

MCF-7, human breast adenocarcinoma cells; Hep-G2, human hepatocellular carcinoma cells.

**Table 4:** Antifungal activity of the *Nepeta juncea* extracts.

Solvent	Plant Part	*Candida albicans*		*C. glabrata*	
		MIC (µg/mL)	MFC (µg/mL)	MIC (µg/mL)	MFC (µg/mL)
Methanol	Leaves	25	50	50	50
	Flowers	50	50	50	100
	Roots	50	100	100	100
Ethanol	Leaves	50	100	50	100
	Flowers	100	100	100	200
	Roots	100	200	200	200
Water	Leaves	100	200	100	200
	Flowers	200	200	200	-
	Roots	200	-	200	-
Fluconazole		25	25	25	25

MIC, minimum inhibitory concentration; MFC, minimum fungicidal concentration.

**Table 5:** Minimum inhibitory concentration (MIC) and minimum bactericidal concentration (MBC) values of the *Nepeta juncea* extracts against the tested bacteria.

Solvent	Plant Part	*Staphylococcus aureus*		*Bacillus cereus*		*Escherichia coli*		*Shigella flexneri*	
		MIC (µg/mL)	MBC (µg/mL)	MIC (µg/mL)	MBC (µg/mL)	MIC (µg/mL)	MBC (µg/mL)	MIC (µg/mL)	MBC (µg/mL)
Methanol	Leaves	25	50	25	50	50	100	50	100
	Flowers	25	50	25	50	50	100	50	100
	Roots	50	100	50	100	100	200	100	200
Ethanol	Leaves	50	100	50	100	100	200	100	200
	Flowers	50	100	50	100	100	200	100	200
	Roots	100	200	100	200	200	-	200	-
Water	Leaves	50	100	50	100	100	200	100	200
	Flowers	100	200	100	200	200	-	200	-
	Roots	100	200	100	200	200	-	200	-
